# SAGE: String-overlap Assembly of GEnomes

**DOI:** 10.1186/1471-2105-15-302

**Published:** 2014-09-15

**Authors:** Lucian Ilie, Bahlul Haider, Michael Molnar, Roberto Solis-Oba

**Affiliations:** Department of Computer Science, University of Western Ontario, N6A 5B7 London, Ontario Canada

**Keywords:** De novo genome assembly, DNA sequencing, String-overlap graph

## Abstract

**Background:**

De novo genome assembly of next-generation sequencing data is one of the most important current problems in bioinformatics, essential in many biological applications. In spite of significant amount of work in this area, better solutions are still very much needed.

**Results:**

We present a new program, SAGE, for de novo genome assembly. As opposed to most assemblers, which are de Bruijn graph based, SAGE uses the string-overlap graph. SAGE builds upon great existing work on string-overlap graph and maximum likelihood assembly, bringing an important number of new ideas, such as the efficient computation of the transitive reduction of the string overlap graph, the use of (generalized) edge multiplicity statistics for more accurate estimation of read copy counts, and the improved use of mate pairs and min-cost flow for supporting edge merging. The assemblies produced by SAGE for several short and medium-size genomes compared favourably with those of existing leading assemblers.

**Conclusions:**

SAGE benefits from innovations in almost every aspect of the assembly process: error correction of input reads, string-overlap graph construction, read copy counts estimation, overlap graph analysis and reduction, contig extraction, and scaffolding. We hope that these new ideas will help advance the current state-of-the-art in an essential area of research in genomics.

**Electronic supplementary material:**

The online version of this article (doi:10.1186/1471-2105-15-302) contains supplementary material, which is available to authorized users.

## Background

Next-generation sequencing (NGS) technologies are causing an unprecedented revolution in biological sciences. The ability to obtain the genome sequence of a species quickly and at a relatively low cost has tremendous biological applications to cancer research, genetic disorders, disease control, neurological research, personalized medicine, etc. NGS technologies such as Illumina, 454, APG, Helicos, Pacific Biosciences, and Ion Torrent [1] produce huge outputs at ever decreasing costs, enabling ambitious projects such as the Genome 10 K Project, [2] (http://www.genome10k.org) whose goal is to obtain the genomes of 10,000 vertebrate species, the 1000 Genomes Project, [3] (http://www.1000genomes.org) that proposes to obtain the genomes of 1000 genetically varying humans, or the Human Microbiome project, [4] (commonfund.nih.gov/Hmp) whose aim is to characterize the microbial communities found at several different sites on the human body.

NGS machines produce short pieces of DNA, called reads, that often need to be assembled together into longer sequences. The ability to de novo assemble genomic data is crucial for the success of many applications including gene expression analysis, structural variation detection, and metagenomics, [5]. There is increased demand in both computing power and algorithmic ideas in order to cope with the increasingly popular NGS data. Great work has been done on creating improved assembly programs, e.g., [6–15], as well as surveying various techniques, [5], or critically and thoroughly evaluating the existing assemblers [16, 17]; we refer the reader to the latter three papers for references to other genome assembly programs or related work. In spite of considerable advances, much improvement is still needed in the current state-of-the-art technology for de novo genome assembly [18].

Reads that overlap significantly offer a good indication that they may come from the same region of the genome. Two main approaches are used in building assemblies. Both use overlaps between the reads but in different ways. In the string-overlap graph approach [19, 20] sufficiently long overlaps between reads are used as edges to connect vertices that represent reads. In the de Bruijn graph approach [21, 22] reads are broken into *k*-mers that are used as vertices connected by edges representing overlaps of length *k*−1. However, regardless of the model, the problems underlying both approaches can be shown to be NP-hard [23]. The de Bruijn graph approach seems counterintuitive, as it breaks the reads into shorter *k*-mers, which appears contrary to the technological efforts to produce longer reads. Nevertheless, most of the top assemblers to date use the de Bruijn graph approach.

We propose a new assembler, SAGE (String-overlap Assembly of GEnomes), that is string-overlap graph based. SAGE includes innovations in almost every aspect of the assembly process: error correction of input reads, string-overlap graph construction, read copy counts estimation, overlap graph analysis and reduction, contig extraction, and scaffolding. We have tested SAGE on short and medium-size genomes against several of the very best assemblers, ABySS [9], SGA [12], SOAPdenovo2 [11], and SPAdes [14] and showed that it performs very well.

## Implementation

The algorithms used in SAGE are described here. Some of the existing ideas are included as well in order to give a complete description.

### Error correction

All NGS datasets contain errors that make any usage of such data, and genome assembly in particular, very difficult. We have used a new program, RACER [24], that consistently exceeds the error correcting performance of existing programs. All datasets have been corrected with RACER before being assembled with SAGE.

### Bidirected graph

Assume a dataset of *n* input reads of length *ℓ* each, sequenced from a genome of length *L*. The string-overlap graph [20] has the reads as vertices. There is an edge between two vertices if there is an overlap between the sequences of the reads in the vertices (or their reverse complements) of length higher than a given threshold, *M*, the minimum overlap size. In order to avoid the complication due to double strandedness of DNA, [25] introduced the bidirected overlap graph, where a read and its reverse complement are represented by the same vertex and an edge has an orientation at each end point, depending on whether the read or its reverse complement is used in producing the overlap defining the edge. Three possible types of edges are thus obtained. Each edge has a string associated with it, obtained from the strings of the reads according to their overlaps. For instance, two strings *xy* and *yz* (*y* is the overlap) produce the string *xyz* for the edge. Assuming no errors in the reads, a consistent path through the graph (for each vertex, the orientation of the ingoing edge must match the orientation of the outgoing edge) spells a substring of the genome. That is also the way of associating a string with a path in the graph.

### String-overlap graph construction

In order to efficiently find all overlaps of length at least *M* between reads, we make the following observation. Whenever two reads share an overlap of length *M* or more bases, there exists a prefix or suffix of length at least *M* in one of the reads that occurs as a substring of the other read. Therefore, we build a hash table with all prefixes and suffixes of all reads (and reverse complements) of length min{64,*M*}. A fast computation of these is enabled by a 2-bit representation of the DNA bases and computation of the prefixes and suffixes as 64-bit integers by fast bit operations.

After the hash table is built, a search is performed for all substrings of length min{64,*M*} of all reads. This is done in one pass through all reads with expected constant time search per substring and fast computation of the next substring, again using efficient bit operations. Each successful search is followed by a fast check for a valid overlap. Whenever an overlap is found, the corresponding edge is inserted in the graph.

### Space-efficient transitive reduction

The string-overlap graph can be very large and a transitive reduction is performed to significantly decrease its size. An edge *e*=(*r*_1_,*r*_2_) is transitive if there is a read *r*_3_ and edges *e*_1_=(*r*_1_,*r*_3_),*e*_2_=(*r*_3_,*r*_2_) such that the string of the edge *e* is the same as the one of the path (*e*_1_,*e*_2_). Noticing that the overlaps producing the edges *e*_1_ and *e*_2_ are longer, and thus more reliable, than the one producing *e*, the transitive edge *e* can be eliminated.

Myers [20] gave a linear expected time algorithm for transitive reduction. While Myers’ algorithm is very efficient, the graph has to be built before being reduced, thus creating a space bottleneck. We have modified Myers’ algorithm to reduce the graph as it is being built. Myers’ essential observation is that the edges adjacent to a vertex have to be considered in increasing order of their lengths. We maintain this order and in addition attempt to reduce the graph as locally as possible. That is, we build only the part of the graph necessary to determine the transitively reducible edges for a given vertex *v*. These edges are marked for elimination but not yet removed. Once all vertices whose transitive reduction can be influenced by the edges incident with *v* have been investigated, the edges of *v* marked for elimination can be removed, thus reducing the space during construction. The running time for building the transitively reduced graph remains the same as if the complete string-overlap graph is first constructed and then transitive edges are removed, however the space decreases very much. This is essential for the entire SAGE algorithm since graph construction is the most space consuming step.

### Graph simplification

The next step is further simplifying the graph. First, any path consisting exclusively of vertices of indegree and outdegree one is compressed to a single edge, subsequently called composite edge; edges that are not composite are called simple. The string spelled by the composite path is stored with the new edge along with the information concerning the reads corresponding to the collapsed vertices.

Our error correction procedure is very effective, however, errors remain in the corrected dataset. The correcting step does not remove any reads in order to avoid breaks in coverage. Overlaps between a correct read and one containing errors most likely result in short “dead-end” paths in the graph. Composite path compression might produce dead-end paths consisting of single edges. Whenever the number of reads in one such edge is lower than an experimentally determined threshold, the edge is removed.

Sometimes such erroneous paths can connect back into the graph, resulting in “bubbles”. A bubble is the event of two disjoint single paths between two vertices such that their strings are highly similar but their number of reads is very different. In such a case, if one of the two paths has much lower coverage, then it is removed.

### Egde multiplicity

Assuming complete coverage and no errors, the genome would be represented as the string corresponding to a path in the graph. The number of times an edge is traversed by this path equals the number of times the string associated with the edge occurs in the genome. Myers [20] introduced the A-statistics to identify unique, or single-copy, edges. We generalize these statistics in order to be able to obtain accurate estimates of the number of occurrences of the string associated with each edge in the genome.

Given an edge *e* containing *k* reads such and whose associated string has length *d*, assuming the reads are sampled uniformly from the genome the probability that *e* has multiplicity *m*, that is, its string occurs *m* times in the genome, is given in (1).
1

In order to estimate the actual number of copies *e* has in the genome, we define the logarithm of the ratio between the probability of *e* having *m* or *m*+1 copies; see (2).
2

For *m*=1, this log-odds ratio gives the A-statistics of Myers [20].

### Genome and insert size estimation

When defining the edge multiplicity probability and log-odds ratios, the genome length *L* is necessary but unknown. The values R(*e*,1) are used to estimate *L* using the bootstrap algorithm of Myers [20]. The arrival rate for a unique edge of length *d* and *k* reads is expected to be close to that of the entire genome, which gives the estimate: . Edges of length 1000 or more are initially assumed unique. Subsequent estimates of *L* are used for computing the R(*e*,1) ratios and only those edges with R(*e*,1)≥20 are kept as unique in future iterations. This bootstrap procedure is repeated until the set of unique edges does not change. In practice this happened in five iterations or less and a good estimate for *L* was obtained; see Table 1.

**Table 1:** **Estimated genome sizes for the datasets in Table** 3

Dataset	Actual size	Estimated size	Difference (%)
1	4,215,606	4,225,613	0.24%
2	1,042,519	1,353,267	29.81%
3	2,190,731	2,210,896	0.92%
4	1,892,775	1,887,639	-0.27%
5	4,277,185	4,717,507	10.29%
6	2,343,476	2,342,227	-0.05%
7	4,639,675	4,681,050	0.89%
8	3,843,301	4,204,996	9.41%
9	100,286,070	107,544,824	7.24%

Often the dataset does not include information concerning the insert size, that is, the distance between the two reads of the same mate pair. The mean, *μ*, and standard deviation, *σ*, of the insert size distribution are estimated by considering only those mate pairs that belong to the same edge in the string-overlap graph where the distance between the reads in the edge is known.

### Maximum likelihood assembly

Assembling a genome as the shortest string that contains the given reads suffers from an important drawback, that of overcompressing the genome or overcollapsing the repeats. This was already noticed by Myers [19], where the maximum likelihood reconstruction of the genome was proposed, that is, instead of the shortest genome, the one that is most likely to have produced the genome is searched for. Medvedev and Brudno [26] considered a very interesting approach to maximum likelihood assembly that is suitable for our purpose.

Our goal is to produce good estimates for the read copy counts, that is, for each read, the number of times its sequence appears in the genome. For a read *r*_*i*_, assume its copy count is *c*_*i*_. The *c*_*i*_ values are unknown. What is known are their observed values, say *x*_*i*_, and the likelihood, , that needs to be maximized, shown in (3).
3

Maximizing  is the same as minimizing its negative logarithm, see (4).
4

where *C* is a constant independent of the *c*_*i*_’s.

For each *i*, the path in the graph spelling the genome sequence traverses the edge containing the read *r*_*i*_ exactly *c*_*i*_ times. Since  is separable convex, maximizing the likelihood is reduced to a convex min-cost bidirected flow problem in a network build on the string-overlap graph. First, each convex cost function is given a three-piece linear approximation. Then the bidirected flow problem is reduced to a directed flow problem that is solved using the CS2 algorithm [27] downloaded from http://www.igsystems.com/cs2. We refer to [26] for details.

### Read copy counts estimation

As explained in the previous section, the solution to the bidirected flow problem gives an estimation of the copy counts *c*_*i*_’s of the reads. Crucial are the bounds we set on the capacities of the edges. For vertices, we must set a lower bound of 1 since the reads in vertices must be included in the assembly. For edges, we use the R(*e*,*m*) statistics that we introduced previously. We consider only long edges (1000 bp or more) for which the statistics should work well. For one such edge *e*, if R(*e*,1)≥*T*, for some threshold *T* (*T*=3 works well in practice), then we set the lower and upper bounds on the capacity of the edge as *l*(*e*)=*u*(*e*)=1. If R(*e*,1)<3, then we find the smallest *m* such that R(*e*,*m*−1)≤−*T* and R(*e*,*m*)≥*T* and set the lower bound *l*(*e*)=*m* and the upper bound to some large value, *u*(*e*)=*∞*. In case the above procedure fails to assign lower bounds, we set *l*(*e*)=1 and *u*(*e*)=*∞*. For the composite edges shorter than 1000 bp but containing at least 30 reads, we assign *l*(*e*)=1 and *u*(*e*)=*∞*. The remaining edges receive the trivial *l*(*e*)=0, *u*(*e*)=*∞*.

The estimation of the copy counts obtained using the above procedure is very good. Table 2 gives the comparison between our procedure and the one of [26]. The latter one works well for synthetic datasets, as reported in [26], but not for real data.

**Table 2:** **Predicted read copy count comparison for the datasets in Table** 3

Dataset	MB09	SAGE
1	3.19	**82.71**
2	4.85	**71.87**
3	7.24	**59.91**
4	9.37	**58.61**
5	5.31	**65.90**
6	9.40	**56.26**
7	-	**65.40**
8	-	**66.67**
9	-	**73.22**

Predicted read copy count comparison between the algorithm of [26], denoted MB09, and the procedure used by SAGE. The values given are the percentages of correctly predicted copy counts. The MB09 algorithm could not process the last 3 datasets from Table 3.

### Further graph simplification

Based on the flow computed above, several further simplifications are performed to the graph. For a vertex, *r*, with only one incoming edge, (*s*,*r*) and outgoing edges (*r*,*r*_*i*_),1≤*i*≤*k*, we remove the vertex *r* and its adjacent edges and add edges (*s*,*r*_*i*_), 1≤*i*≤*k*. The flow on the edge (*s*,*r*_*i*_) is the same as it was on the edge (*r*,*r*_*i*_). A similar modification is done for vertices with only one outgoing edge.

For a vertex *r* that has a self loop (*r*,*r*) and only two adjacent edges, (*s*,*r*) and (*r*,*t*), we remove the vertex *r* and its edges and replace them with (*s*,*t*). Note that the flow on (*s*,*t*) is the same as it was on either (*s*,*r*) or (*r*,*t*).

### Mate pair support

A vertex having more than one incoming edge and more than one outgoing edge is “ambiguous”. Mate pairs are used in connection with the flow to solve some of these ambiguities. For a vertex *r*, an incoming edge (*s*,*r*) and an outgoing edge (*r*,*t*), we say that a mate pair (*r*_1_,*r*_2_) supports the path (*s*,*r*,*t*) through *r* if all paths of length within the range *μ*±3*σ* from *r*_1_ to *r*_2_ include the path (*s*,*r*,*t*). Note that this is significantly more general than simply having *r*_1_ on the edge (*s*,*r*) and *r*_2_ on the edge (*r*,*t*). There may be many paths of length *μ*±3*σ* between *r*_1_ and *r*_2_.

The support required in SAGE for merging a pair of adjacent edges is at least 5. When edges (*s*,*r*) and (*r*,*t*) are merged, an edge (*s*,*t*) is added with flow equal to the minimum flow on (*s*,*r*) and (*r*,*t*). The edge with lower flow out of (*s*,*r*) and (*r*,*t*) is then deleted and the other has its flow decreased by the minimum of the two. If both have the same flow, then both are deleted. Often, *r* has only four adjacent edges and so it is completely resolved by this procedure.

In order to save space, we compute the paths of length up to *μ*+3*σ* from each node and then consider the reads on all edges incident to that node.

Due to lack of coverage in some regions or errors in the reads, some mate pairs may have no path to connect them in the graph. We can still use their support in such cases as follows. Consider a mate pair (*r*_1_,*r*_2_) such that *r*_1_ belongs to the edge *e*_1_ and *r*_2_ belongs to the edge *e*_2_. If the sum of the distances from *r*_1_ to the end of the edge *e*_1_ and from the corresponding end of *e*_2_ to *r*_2_ is less than *μ*+3*σ*, then (*r*_1_,*r*_2_) supports the edges (*e*_1_,*e*_2_) merging. This type of support from mate pairs is less reliable and we strengthen it by considering only edges with non-zero flow or having sufficiently long associated paths (at least 100 bp).

Assuming sufficient support is accumulated to merge the edges *e*_1_ and *e*_2_, the distance between their ends is estimated based on *μ*. If they overlap, there will be no gap, otherwise the gap is filled with N’s.

Finally, the set of output contigs consists of the strings of minimum required length (default is 100) that are associated with edges of non-zero flow.

## SAGE overview

We present here a brief overview of the main stages of SAGE using all the procedures presented above.


## Results and discussion

### Assemblers

We have chosen for comparison several leading de novo assembly programs, according to their ranking by the Assemblathon 1 competition [16] and the GAGE survey [17] which rank ALLPATHS [8] and SOAPdenovo [10], both de Bruijn graph based, at the top. We have tested against the improved SOAPdenovo2 [11], but we could not use ALLPATHS since it is unable to assemble single-library real datasets. We have also included ABySS [9] as one of the most widely used and frequently updated programs and SPAdes [14] as one of the best new assemblers for bacterial genomes [28], both de Bruijn graph based. Our comparison is by no means exhaustive. The main point is to show that SAGE is competitive.

The most notable exception from the domination of the de Bruijn graph-based assemblers is the recent SGA program [12], which is string-overlap graph based. SGA uses compressed data structures to keep the memory requirements low, with the main aim of being able to run on low-end computing clusters. Nevertheless, SGA is a successful assembler, ranked third in the Assemblathon 1 competition [16]. Our efforts are complementary to those of [12] in the attempt of producing better assemblers that are string-overlap graph based.

### Datasets

We compare the assemblers on real datasets only. We have downloaded a number of datasets from the NCBI web site (http://www.ncbi.nlm.nih.gov), with varied read length and genome size, together with a *C.elegans* dataset, from http://www.wormbase.org, that has been previously used by [12]. As noticed by [12], the genome of *C.elegans* is an example of an excellent test case for assembly algorithms due to its long (100 MB) and accurate reference sequence, free of SNPs and structural variants. The accession numbers for all datasets and their reference genomes are given in Table 3, together with all their parameters.

**Table 3:** **The datasets used for evaluation**

Dataset	Organism	Accession	Reference	Genome	Read	Number	Number of	Coverage
		number	genome	length	length	of reads	base pairs	
1	*Bacillus subtilis*	DRR000852	NC_000964.3	4,215,606	75	3,519,504	263,962,800	62.62
2	*Chlamydia trachomatis*	ERR021957	NC_000117.1	1,042,519	37	7,825,944	289,559,928	277.75
3	*Streptococcus pseudopneumoniae*	SRR387784	NC_015875.1	2,190,731	100	4,407,248	440,724,800	201.18
4	*Francisella tularensis*	SRR063416	NC_006570.2	1,892,775	101	6,907,220	697,629,220	368.57
5	*Leptospira interrogans*	SRR397962	NC_005823.1	4,277,185	100	7,127,250	712,725,000	166.63
6	*Porphyromonas gingivalis*	SRR413299	NC_002950.2	2,343,476	100	9,497,946	949,794,600	405.29
7	*Escherichia coli*	SRR072099	NC_000913.2	4,639,675	36	30,355,432	1,092,795,552	235.53
8	*Clostridium thermocellum*	SRR400550	NC_009012.1	3,843,301	36	31,994,160	1,151,789,760	299.69
9	*Caenorhabditis elegans*	SRR065390	WS222	100,286,070	100	67,617,092	6,761,709,200	67.42

The datasets are sorted increasingly by the total number of base pairs. All datasets and reference genome sequences are obtained from the NCBI, except C.elegans that is from http://www.wormbase.org.

### Evaluation method

The most recent version of each assembler has been used and each program was run for all possible *k*-mers or minimum overlap sizes on each dataset. The *k*-mer or minimum overlap size producing the highest scaffold N50 value is reported. N50 is representative for the quality of the assembly, however producing high values of N50 is not sufficient by itself, as it says nothing of the quality of the contigs. In particular, misassembled contigs can artificially increase N50. Therefore, we have used the QUAST comprehensive evaluation tool [29] to compare the quality of the assemblies.

The most relevant parameter is NGA50, which is computed by aligning the contigs to the reference genome, splitting them at misassembly breakpoints, eliminating unaligned parts, and then computing the N50 of the obtained contigs with respect to the length of the reference genome.

We present also the NGA75, the length of the largest alignment, the fraction of genome covered, the number of unaligned contigs, the average number of indels and mismatches for each 100 kbp, and the number of (local) misassemblies as the most important parameters computed by QUAST. All the information given by QUAST is included in the Additional file 1. Details on how each program was run and its output was evaluated by QUAST, including the precise commands used, are also given in the Additional file 1.

### Comparison

The best assemblies produced by all the assemblers considered are compared and presented in Tables 4, 5, 6, 7, 8, 9, 10, 11. Whenever meaningful, we present also the average of the results. SAGE has the best NGA50, NGA75, and length of the longest aligned contig for most datasets. For NGA50, the average of SAGE is 50% better than the second one, from SPAdes. For genome coverage, SAGE and ABySS are tied for the first place. ABySS has the lowest number of unaligned contigs for the bacterial datasets but the highest for *C.elegans* for which SAGE performed the best. SGA produces the fewest misassemblies, followed closely by SOAPdenovo2. For local misassemblies, SGA is the best. Overall, SGA produces the fewest errors but also the lowest NGA50, NGA75, and longest contig values. Concerning the average number of indels and mismatches per 100 kbp, significant differences are seen between datasets, with 2, 3, 4 having many more errors than the other ones. All assemblers produce similar values with ABySS, SGA and SAGE at the top, with almost the same number of errors.

**Table 4:** **NGA50 comparison; best results in bold**

NGA50	ABySS	SGA	SOAP2	SPAdes	SAGE
1	423,890	68,419	551,507	441,472	**924,197**
2	301,840	97,593	225,668	**696,260**	669,089
3	23,245	21,876	26,356	18,167	**30,232**
4	**25,749**	23,314	23,294	23,762	23,961
5	117,711	83,128	132,993	38,735	**182,864**
6	35,564	37,013	42,835	32,926	**54,125**
7	**101,741**	10,038	98,665	36,300	96,980
8	52,944	23,747	54,744	52,142	**54,883**
9	18,210	20,436	31,973	20,468	**32,442**
Avg.	122,322	42,840	132,004	151,137	**229,864**

**Table 5:** **NGA75 comparison; best results in bold**

NGA75	ABySS	SGA	SOAP2	SPAdes	SAGE
1	162,208	40,124	306,202	**306,452**	306,386
2	160,704	51,570	125,082	**339,321**	307,765
3	9,847	7,570	6,785	6,052	**10,040**
4	**14,491**	13,117	13,117	13,638	13,377
5	58,556	40,333	64,594	22,071	**87,232**
6	20,005	18,062	19,982	15,716	**25,176**
7	**56,943**	5,270	54,790	18,706	54,784
8	28,805	8,618	25,243	25,255	**29,529**
9	7,126	7,596	13,232	8,122	**14,095**
Avg.	57,632	21,362	69,892	83,926	**94,265**

**Table 6:** **Max alignment; best results in bold**

Max	ABySS	SGA	SOAP2	SPAdes	SAGE
1	800,991	241,307	1,014,436	**1,037,023**	1,016,322
2	359,339	210,791	339,457	**696,260**	669,089
3	**125,616**	**125,616**	125,563	74,151	**125,616**
4	87,729	87,426	87,417	87,801	**87,862**
5	413,583	319,895	320,270	137,901	**550,746**
6	**172,567**	167,699	167,686	154,317	172,565
7	326,073	54,214	325,634	162,291	**326,332**
8	186,547	106,016	186,433	**195,919**	186,424
9	213,835	239,959	382,096	171,314	**383,476**
Avg.	298,476	172,547	327,666	301,886	**390,937**

**Table 7:** **Genome coverage (%); best results in bold**

Coverage	ABySS	SGA	SOAP2	SPAdes	SAGE
1	**99.04**	98.67	98.63	98.63	98.95
2	98.57	98.04	94.65	99.36	**99.49**
3	**83.30**	82.82	81.54	82.01	83.19
4	**95.61**	93.07	92.58	93.80	93.56
5	99.49	98.77	98.75	95.15	**99.67**
6	**97.97**	95.08	95.62	95.95	97.77
7	**95.62**	94.10	94.80	95.09	95.42
8	95.78	92.63	92.81	94.08	**95.85**
9	95.49	95.19	95.28	95.48	**96.94**
Avg.	**95.65**	94.26	93.85	94.40	**95.65**

**Table 8:** **Unaligned contigs; best results in bold**

Bad contigs	ABySS	SGA	SOAP2	SPAdes	SAGE
1	1	**0**	8	3	**0**
2	**10**	16	16	**10**	19
3	**3**	26	29	30	14
4	**0**	1	2	11	1
5	**0**	1	**0**	266	**0**
6	**0**	**0**	**0**	423	**0**
7	**1**	4	**1**	2	2
8	1	**0**	11	1	3
9	978	304	272	363	**267**

**Table 9:** **Misassemblies; best results in bold**

Indel/mm	ABySS	SGA	SOAP2	SPAdes	SAGE
1	1	**0**	**0**	1	10
2	**1**	**1**	**1**	**1**	**1**
3	149	**108**	118	115	169
4	94	35	34	28	**26**
5	8	**2**	7	114	11
6	10	**5**	15	8	17
7	26	**4**	5	22	25
8	18	**1**	9	6	32
9	1399	**144**	246	713	1196

**Table 10:** **Local misassembles; best results in bold**

Indel/mm	ABySS	SGA	SOAP2	SPAdes	SAGE
1	**0**	**0**	13	1	11
2	11	**5**	41	9	26
3	100	87	85	**78**	96
4	**46**	**46**	47	**46**	56
5	29	42	58	159	**28**
6	11	17	40	**10**	14
7	80	**1**	71	17	83
8	9	**0**	69	6	164
9	6282	2209	2703	**839**	1474

**Table 11:** **Average number of indels and mismatches per 100 kbp; best results in bold**

Indel/mm	ABySS	SGA	SOAP2	SPAdes	SAGE
1	8.65	**2.18**	7.14	5.78	5.61
2	825.09	833.32	**817.64**	834.67	893.48
3	2407.49	2417.37	2387.42	**2368.18**	2402.27
4	518.38	522.02	527.28	509.66	**530.76**
5	19.01	18.89	45.86	581.17	**15.58**
6	**10.80**	35.81	13.83	22.33	26.75
7	36.20	**8.82**	29.77	17.77	47.86
8	14.45	**3.15**	73.19	28.18	46.04
9	50.08	**34.33**	34.84	76.92	47.63

The time and space comparison is presented in Table 12. In order to facilitate comparison we present the time (seconds) and space (megabytes) per input mega base pairs. This way we can also compute averages. ABySS uses the least amount of space and SOAPdenovo2 is the fastest, with SAGE coming closely in second place for both time and space. Actual time and space values are presented in the Additional file 1.

**Table 12:** **Time and space comparison**

Data	ABySS	SGA	SOAP2	SPAdes	SAGE
1	2.07 / 3.77	11.14 / 8.37	0.68 / 17.15	6.79 / 5.92	**0.53** / **3.00**
2	2.56 / 7.75	13.99 / 12.00	**0.55** / 22.04	12.26 / 22.88	0.57 / **3.03**
3	1.03 / **1.02**	12.87 / 9.23	**0.36** / 15.20	6.68 / 2.98	0.37 / 1.78
4	1.41 / **1.08**	13.34 / 9.77	0.53 / 13.68	6.67 / 1.74	**0.34** / 1.45
5	1.13 / **0.99**	13.69 / 9.87	**0.34** / 10.94	7.03 / 3.11	0.43 / 1.90
6	2.05 / **1.87**	12.69 / 13.32	0.52 / 9.52	5.43 / 3.73	**0.51** / 3.25
7	1.78 / **1.63**	11.03 / 11.57	0.45 / 8.28	4.72 / 3.24	**0.44** / 2.83
8	2.57 / 1.40	4.21 / 4.45	**0.36** / 6.62	2.88 / **0.91**	3.04 / 5.98
9	3.99 / **2.22**	19.88 / 9.90	1.47 / 4.94	19.72 / 7.17	**0.81** / 2.54
Avg.	2.07 / **2.41**	12.54 / 9.83	**0.58** / 12.04	8.02 / 5.74	0.78 / 2.86

The results are presented in the format “time/space” with the time in seconds and space in megabytes, both per input mega base pairs. The best results are shown in bold. The last row gives the average values.

## Conclusions

Myers [20] suggests that string-overlap graph based assemblers should perform better than those based on the de Bruijn graph and our work aims at supporting his prediction.

SAGE builds upon great existing work and brings several new ideas, such as the efficient computation of the transitive reduction of the string overlap graph, the use of (generalized) edge multiplicity statistics for improved estimation of copy counts, and the improved use of mate pairs and flow for supporting edge merging.

We believe that our work shows that the potential of string-overlap graph-based assemblers is higher than previously thought. SAGE is currently able to successfully handle short and medium-size genomes but future versions will handle mammalian genomes as well. Also, we plan to work on reducing the number of misassemblies produced by SAGE.

We hope that some of the ideas presented will be used also by others in order to boost the development of this type of assemblers and further improve the current state-of-the-art. As read length is going to grow, we expect that string-overlap graph-based assemblers will have a better chance to improve.

### Data access

All datasets are available from the NCBI, except *C.elegans* which is from http://www.wormbase.org. The genome assemblers are available as follows: ABySS at http://www.bcgsc.ca/platform/bioinfo/software/abyss, SGA at github.com/jts/ sga, SOAPdenovo2 at soapdenovo2.sourceforge.net/, and SPAdes at bioinf.spbau.ru/spades. The CS2 program is available at http://www.igsystems.com/cs2.

## Availability and requirements

**Project name:** SAGE**Project home page:**http://www.csd.uwo.ca/~ilie/SAGE/

**Operating system(s):** Platform independent

**Programming language:** C++

**Other requirements:** none

**License:** GNU

**Any restrictions to use by non-academics:** none

## Electronic supplementary material

Additional file 1: **The supplementary material includes all the details on how each program was run and its output was evaluated by QUAST, including the precise commands used.** All details for evaluating the assemblies, as given by QUAST, are also included, as well as the actual time and space values. (PDF 306 KB)
