# Supplementary material for: SAGE: String-overlap Assembly of GEnomes
Source: BMC Bioinformatics. 2014 Sep 15;15(1):302. doi: 10.1186/1471-2105-15-302 (PMC4174676; doi:10.1186/1471-2105-15-302)
Supplement: Supplementary file 1 — Additional file 1: The supplementary material includes all the details on how each program was run and its output was evaluated by QUAST, including the precise commands used. All details for evaluating the assemblies, as given by QUAST, are also included, as well as the actual time and space values. (PDF 306 KB) [file 12859_2014_6583_MOESM1_ESM.pdf]

SAGE: String-overlap Assembly of GEnomes

Lucian Ilie, Bahlul Haider, Michael Molnar, and Roberto Solis-Oba

— Supplementary material —

Details on running the programs

ABySS version 1.3.4 was used with the following command:

`abyss-pe k=`[k-mer length] `name=`[output name] `in=`[input file]

SGA version 0.10.10 was used with the following commands:

```
sga preprocess -o [output file] [input file]
sga index -a ropebwt [input file]
sga correct -k [k-mer length] -o [output file] [input file]
sga index -a ropebwt [input file]
sga filter -x 2 [input file]
sga fm-merge -m [minimum overlap length] -o [output file] [input file]
sga index -d 1000000 [input file]
sga rmdup [input file]
sga overlap -m [minimum overlap length] [input file]
sga assemble -m [overlap length] -g 0 -r 10 -o [output file] [input file]
sga-align [contig file] [input file]
sga-bam2de.pl -n 5 [input file]
sga-astat.py -m 200 [input file] > [output file]
sga scaffold -m 200 --pe [paired-end file] -a [a-statistic file] -o [output file] [contig file]
sga scaffold2fasta -m 200 -a [graph file] -o [output file] -d 1 --use-overlap --write-unplaced [input file]
```

SOAPdenovo2 version 2.04 was used with following command:

`SOAPdenovo all -s` [configuration file] `-K` [k-mer length] `-p 1 -o` [output file]

SPAdes version 3.1.0 was used with the following command:

`spades.py -1` [forward reads] `-2` [reverse reads] `-t` [number of threads] `-o` [output directory]

SAGE was used with following command:

`sage` [input file(s)] [output file] [min overlap]

QUAST was used with the following command:
`quast.py --min-contig 200 -o` [output name] `-R` [reference file] [scaffold files]

Complete comparison results

The complete comparison using QUAST is presented in the following pages.

Dataset	Accession #	Read Length	Per-Base Error	Reference Genome	Genome Length	Number of Reads	Total bp	Coverage
B.subtilis	DRR000852	75	0.58%	NC_000964.3	4,215,606	3,519,504	263,962,800	62.62
C.trachomatis	ERR021957	37	3.90%	NC_000117.1	1,042,519	7,825,944	289,559,928	277.75
S.pseudopneumoniae	SRR387784	100	1.06%	NC_015875.1	2,190,731	4,407,248	440,724,800	201.18
F.tularensis	SRR063416	101	0.60%	NC_006570.2	1,892,775	6,907,220	697,629,220	368.57
L.interrogans	SRR397962	100	0.21%	NC_005823.1	4,277,185	7,127,250	712,725,000	166.63
P.gingivalis	SRR413299	100	0.23%	NC_002950.2	2,343,476	9,497,946	949,794,600	405.29
E.coli	SRR072099	36	0.87%	NC_000913.2	4,639,675	30,355,432	1,092,795,552	235.53
C.thermocellum	SRR400550	36	2.91%	NC_009012.1	3,843,301	31,994,160	1,151,789,760	299.69
C.elegans	SRR065390	100	0.35%	c_elegans	100,286,070	67,617,092	6,761,709,200	67.42

Dataset	Exact search		BWA		
	bp mapped	% mapped	bp mapped	reads mapped	% mapped
B.subtilis	170,408,550	64.56%	251,541,675	3,353,889	95.29%
C.trachomatis	66,376,631	22.92%	129,671,532	3,504,636	44.78%
S.pseudopneumoniae	152,253,200	34.55%	408,639,600	4,086,396	92.72%
F.tularensis	381,587,393	54.70%	691,743,344	6,848,944	99.16%
L.interrogans	578,226,000	81.13%	661,384,300	6,613,843	92.80%
P.gingivalis	752,283,200	79.20%	945,429,000	9,454,290	99.54%
E.coli	796,578,120	72.89%	887,065,884	24,640,719	81.17%
C.thermocellum	397,718,244	34.53%	415,224,504	11,534,014	36.05%
C.elegans	4,768,401,900	70.52%	6,492,212,600	64,922,126	96.01%

NGA50	ABySS	SGA	SOAP2	SPAdes	SAGE
B.subtilis	423,890	68,419	551,507	441,472	**924,197**
C.trachomatis	301,840	97,593	225,668	**696,260**	669,089
S.pseudopneumonie	23,245	21,876	26,356	18,167	**30,232**
F.tularensis	**25,749**	23,314	23,294	23,762	23,961
L.interrogans	117,711	83,128	132,993	38,735	**182,864**
P.gingivalis	35,564	37,013	42,835	32,926	**54,125**
E.coli	**101,741**	10,038	98,665	36,300	96,980
C.thermocellum	52,944	23,747	54,744	52,142	**54,883**
C.elegans	18,210	20,436	31,973	20,468	**32,442**
AVERAGE	122,322	42,840	132,004	151,137	**229,864**

NGA75	ABySS	SGA	SOAP2	SPAdes	SAGE
B.subtilis	162,208	40,124	306,202	**306,452**	306,386
C.trachomatis	160,704	51,570	125,082	**339,321**	307,765
S.pseudopneumonie	9,847	7,570	6,785	6,052	**10,040**
F.tularensis	**14,491**	13,117	13,117	13,638	13,377
L.interrogans	58,556	40,333	64,594	22,071	**87,232**
P.gingivalis	20,005	18,062	19,982	15,716	**25,176**
E.coli	**56,943**	5,270	54,790	18,706	54,784
C.thermocellum	28,805	8,618	25,243	25,255	**29,529**
C.elegans	7,126	7,596	13,232	8,122	**14,095**
AVERAGE	57,632	21,362	69,892	83,926	**94,265**

Largest alignment	ABySS	SGA	SOAP2	SPAdes	SAGE
B.subtilis	800,991	241,307	1,014,436	**1,037,023**	1,016,322
C.trachomatis	359,339	210,791	339,457	**696,260**	669,089
S.pseudopneumonie	**125,616**	**125,616**	125,563	74,151	**125,616**
F.tularensis	87,729	87,426	87,417	87,801	**87,862**
L.interrogans	413,583	319,895	320,270	137,901	**550,746**
P.gingivalis	**172,567**	167,699	167,686	154,317	172,565
E.coli	326,073	54,214	325,634	162,291	**326,332**
C.thermocellum	186,547	106,016	186,433	**195,919**	186,424
C.elegans	213,835	239,959	382,096	171,314	**383,476**
AVERAGE	298,476	172,547	327,666	301,886	**390,937**

Genome fraction (%)	ABySS	SGA	SOAP2	SPAdes	SAGE
B.subtilis	**99.04**	98.67	98.63	98.63	98.95
C.trachomatis	98.57	98.04	94.65	99.36	**99.49**
S.pseudopneumonie	**83.30**	82.82	81.54	82.01	83.19
F.tularensis	**95.61**	93.07	92.58	93.80	93.56
L.interrogans	99.49	98.77	98.75	95.15	**99.67**
P.gingivalis	**97.97**	95.08	95.62	95.95	97.77
E.coli	**95.62**	94.10	94.80	95.09	95.42
C.thermocellum	95.78	92.63	92.81	94.08	**95.85**
C.elegans	95.49	95.19	95.28	95.48	**96.94**
AVERAGE	**95.65**	94.26	93.85	94.40	**95.65**

# unaligned contigs	ABySS	SGA	SOAP2	SPAdes	SAGE
B.subtilis	1	**0**	8	3	**0**
C.trachomatis	**10**	16	16	**10**	19
S.pseudopneumonie	**3**	26	29	30	14
F.tularensis	**0**	1	2	11	1
L.interrogans	**0**	1	**0**	266	**0**
P.gingivalis	**0**	**0**	**0**	423	**0**
E.coli	**1**	4	**1**	2	2
C.thermocellum	1	**0**	11	1	3
C.elegans	978	304	272	363	**267**
Total	994	352	339	1109	**306**

# misassemblies	ABySS	SGA	SOAP2	SPAdes	SAGE
B.subtilis	1	**0**	**0**	1	10
C.trachomatis	**1**	**1**	**1**	**1**	**1**
S.pseudopneumonie	149	**108**	118	115	169
F.tularensis	94	35	34	28	**26**
L.interrogans	8	**2**	7	114	11
P.gingivalis	10	**5**	15	8	17
E.coli	26	**4**	5	22	25
C.thermocellum	18	**1**	9	6	32
C.elegans	1399	**144**	246	713	1196
AVERAGE	190	**33**	48	112	165

# local misassemblies	ABySS	SGA	SOAP2	SPAdes	SAGE
B.subtilis	**0**	**0**	13	1	11
C.trachomatis	11	**5**	41	9	26
S.pseudopneumonie	100	87	85	**78**	93
F.tularensis	**46**	**46**	47	**46**	56
L.interrogans	29	42	58	159	**28**
P.gingivalis	11	17	40	**10**	14
E.coli	80	**1**	71	17	83
C.thermocellum	9	**0**	69	6	164
C.elegans	6282	2209	2703	**839**	1474
AVERAGE	730	267	347	**129**	217

mismatch + indels	ABySS	SGA	SOAP2	SPAdes	SAGE
B.subtilis	8.65	**2.18**	7.14	5.78	19.95
C.trachomatis	825.09	833.32	**817.64**	834.67	831.27
S.pseudopneumonie	2407.49	2417.37	2387.42	**2368.18**	2406.82
F.tularensis	518.38	522.02	527.28	509.66	**503.81**
L.interrogans	19.01	18.89	45.86	581.17	**15.81**
P.gingivalis	**10.80**	35.81	13.83	22.33	17.41
E.coli	36.20	**8.82**	29.77	17.77	29.95
C.thermocellum	14.45	**3.15**	73.19	28.18	17.96
C.elegans	50.08	**34.33**	34.84	76.92	60.89

	Time (s)				
	ABySS	SGA	SOAP2	SPAdes	SAGE
**B.subtilis**	546	2,940	180	1,791	**139**
**C.trachomatis**	740	4,050	**158**	3,549	165
**S.pseudopneumonie**	455	5,673	**157**	2,942	163
**F.tularensis**	985	9,307	373	4,651	**239**
**L.interrogans**	807	9,757	**243**	5,012	304
**P.gingivalis**	851	12,963	283	8,042	**268**
**E.coli**	1,947	12,051	492	5,158	**486**
**C.thermocellum**	2,960	4,853	**419**	3,313	3,501
**C.elegans**	26,991	134,412	9,913	133,348	**5,494**

	Time(s/Mbp)				
	ABySS	SGA	SOAP2	SPAdes	SAGE
**B.subtilis**	2.07	11.14	0.68	6.79	**0.53**
**C.trachomatis**	2.56	13.99	**0.55**	12.26	0.57
**S.pseudopneumonie**	1.03	12.87	**0.36**	6.68	0.37
**F.tularensis**	1.41	13.34	0.53	6.67	**0.34**
**L.interrogans**	1.13	13.69	**0.34**	7.03	0.43
**P.gingivalis**	2.05	12.69	0.52	5.43	**0.51**
**E.coli**	1.78	11.03	0.45	4.72	**0.44**
**C.thermocellum**	2.57	4.21	**0.36**	2.88	3.04
**C.elegans**	3.99	19.88	1.47	19.72	**0.81**
**Average**	2.07	12.54	**0.58**	8.02	0.78

	Space (MB)				
	ABySS	SGA	SOAP2	SPAdes	SAGE
**B.subtilis**	996	2,209	4,526	1,562	**792**
**C.trachomatis**	2,244	3,474	6,381	6,626	**878**
**S.pseudopneumonie**	**451**	4,066	6,698	1,314	785
**F.tularensis**	**750**	6,813	9,545	1,216	1,009
**L.interrogans**	**703**	7,036	7,797	2,218	1,357
**P.gingivalis**	**533**	9,396	8,963	3,974	1,165
**E.coli**	**1,777**	12,649	9,046	3,545	3,089
**C.thermocellum**	1,607	5,130	7,624	**1,049**	6,891
**C.elegans**	**15,042**	66,937	33,386	48,511	17,206

	Space(MB/Mbp)				
	ABySS	SGA	SOAP2	SPAdes	SAGE
**B.subtilis**	3.77	8.37	17.15	5.92	**3.00**
**C.trachomatis**	7.75	12.00	22.04	22.88	**3.03**
**S.pseudopneumonie**	**1.02**	9.23	15.20	2.98	1.78
**F.tularensis**	**1.08**	9.77	13.68	1.74	1.45
**L.interrogans**	**0.99**	9.87	10.94	3.11	1.90
**P.gingivalis**	**1.87**	13.32	9.52	3.73	3.25
**E.coli**	**1.63**	11.57	8.28	3.24	2.83
**C.thermocellum**	1.40	4.45	6.62	**0.91**	5.98
**C.elegans**	**2.22**	9.90	4.94	7.17	2.54
**Average**	**2.41**	9.83	12.04	5.74	2.86

B.subtilis	**ABySS**	**SGA**	**SOAP2**	**SPAdes**	**SAGE**
# contigs (>= 0 bp)	89	137	101	49	52
# contigs (>= 1000 bp)	29	96	19	24	16
Total length (>= 0 bp)	4,190,353	4,162,396	4,169,882	4,170,766	4,178,935
Total length (>= 1000 bp)	4,183,291	4,149,193	4,153,022	4,164,264	4,167,141
# contigs	31	137	43	38	40
Total length	4,184,306	4,162,396	4,162,960	4,169,622	4,177,292
Largest contig	800,991	241,307	1,014,549	1,046,888	1,086,665
Reference length	4,215,606	4,215,606	4,215,606	4,215,606	4,215,606
GC (%)	43	43	43	43	43
Reference GC (%)	44	44	44	44	44
N50	423,890	77,429	551,774	441,472	1,021,379
NG50	423,890	68,419	551,774	441,472	1,021,379
N75	162,208	41,567	306,202	306,452	602,762
NG75	162,208	40,124	306,202	306,452	602,762
L50	4	18	3	3	2
LG50	4	19	3	3	2
L75	8	37	5	6	4
LG75	8	38	5	6	4
# misassemblies	1	-	-	1	10
Misassembled contigs length	83,884	-	-	1,148	2,712,488
# local misassemblies	-	-	13	1	11
# unaligned contigs	1 + 0 part	0 + 0 part	8 + 2 part	3 + 0 part	0 + 0 part
Unaligned contigs length	1,105	-	4,980	643	-
Genome fraction (%)	99.04	98.67	98.63	98.63	98.95
Duplication ratio	1.00	1.00	1.01	1.01	1.03
# N's per 100 kbp	23.54	-	101.42	0.00	1.72
# mismatches per 100 kbp	4.67	1.80	0.94	4.14	3.64
# indels per 100 kbp	3.98	0.38	6.20	1.64	1.97
Largest alignment	800,991	241,307	1,014,436	1,037,023	1,016,322
NA50	423,890	77,429	551,507	441,472	924,197
NGA50	423,890	68,419	551,507	441,472	924,197
NA75	162,208	41,567	306,202	306,452	306,386
NGA75	162,208	40,124	306,202	306,452	306,386
LA50	4	18	3	3	3
LGA50	4	19	3	3	3
LA75	8	37	5	6	4
LGA75	8	38	5	6	4

C.trachomatis	**ABySS**	**SGA**	**SOAP2**	**SPAdes**	**SAGE**
# contigs (>= 0 bp)	3,885	41	61	670	65
# contigs (>= 1000 bp)	12	23	13	3	18
Total length (>= 0 bp)	1,283,435	1,041,508	1,060,836	1,094,081	1,072,871
Total length (>= 1000 bp)	1,125,773	1,033,593	1,049,906	1,062,422	1,060,643
# contigs	19	41	30	11	38
Total length	1,129,360	1,041,508	1,056,562	1,066,557	1,069,071
Largest contig	452,436	219,718	478,703	1,038,489	670,146
Reference length	1,042,519	1,042,519	1,042,519	1,042,519	1,042,519
GC (%)	41	41	41	41	41
Reference GC (%)	41	41	41	41	41
N50	301,988	97,635	237,708	1,038,489	670,146
NG50	301,988	97,635	237,708	1,038,489	670,146
N75	160,704	51,999	161,641	1,038,489	311,563
NG75	160,704	51,999	161,641	1,038,489	311,563
L50	2	4	2	1	1
LG50	2	4	2	1	1
L75	3	7	3	1	2
LG75	3	7	3	1	2
# misassemblies	1	1	1	1	1
Misassembled contigs length	452,436	219,718	478,703	1,038,489	28,637
# local misassemblies	11	5	41	9	26
# unaligned contigs	10 + 1 part	16 + 3 part	16 + 1 part	10 + 0 part	19 + 3 part
Unaligned contigs length	23,087	18,371	24,029	28,068	35,552
Genome fraction (%)	98.57	98.04	94.65	99.36	99.49
Duplication ratio	1.08	1.00	1.05	1.00	1.01
# N's per 100 kbp	131.76	-	1,528.54	25.03	113.28
# mismatches per 100 kbp	765.93	770.41	693.39	763.23	755.58
# indels per 100 kbp	59.16	62.91	124.25	71.44	137.90
Largest alignment	359,339	210,791	339,457	696,260	669,089
NA50	301,840	97,593	225,668	696,260	669,089
NGA50	301,840	97,593	225,668	696,260	669,089
NA75	143,144	51,570	125,082	339,321	307,765
NGA75	160,704	51,570	125,082	339,321	307,765
LA50	2	4	2	1	1
LGA50	2	4	2	1	1
LA75	4	7	4	2	2
LGA75	3	7	4	2	2

S.pseudopneumonie	**ABySS**	**SGA**	**SOAP2**	**SPAdes**	**SAGE**
# contigs (>= 0 bp)	232	177	546	315	123
# contigs (>= 1000 bp)	82	85	66	108	65
Total length (>= 0 bp)	2,226,218	2,112,309	2,168,503	2,140,097	2,203,782
Total length (>= 1000 bp)	2,197,826	2,077,654	2,081,837	2,093,239	2,185,902
# contigs	95	177	161	186	97
Total length	2,206,161	2,112,309	2,116,867	2,127,769	2,200,326
Largest contig	203,373	202,327	202,223	114,391	203,300
Reference length	2,190,731	2,190,731	2,190,731	2,190,731	2,190,731
GC (%)	40	40	40	40	40
Reference GC (%)	40	40	40	40	40
N50	56,054	43,415	59,758	29,226	69,356
NG50	56,054	43,415	53,932	28,427	69,356
N75	24,793	20,711	27,200	16,679	33,835
NG75	25,983	18,496	24,367	15,367	33,835
L50	11	14	11	18	9
LG50	11	14	12	19	9
L75	27	31	25	42	20
LG75	26	34	27	45	20
# misassemblies	149	108	118	115	169
Misassembled contigs length	1,930,978	1,545,999	1,710,308	1,536,786	1,933,903
# local misassemblies	100	87	85	78	93
# unaligned contigs	3 + 13 part	26 + 22 part	29 + 25 part	30 + 25 part	14 + 13 part
Unaligned contigs length	53,103	50,325	60,348	97,305	59,506
Genome fraction (%)	83.30	82.82	81.54	82.01	83.19
Duplication ratio	1.19	1.14	1.15	1.13	1.19
# N's per 100 kbp	86.89	89.14	245.98	-	-
# mismatches per 100 kbp	2,327.32	2,329.52	2,291.03	2,293.70	2,310.98
# indels per 100 kbp	80.17	87.85	96.39	74.48	91.29
Largest alignment	125,616	125,616	125,563	74,151	125,616
NA50	23,245	23,245	26,953	18,426	27,048
NGA50	23,245	21,876	26,356	18,167	30,232
NA75	9,296	9,335	8,702	6,988	9,320
NGA75	9,847	7,570	6,785	6,052	10,040
LA50	24	24	22	31	23
LGA50	24	26	23	32	22
LA75	60	59	56	75	55
LGA75	59	66	63	82	54

F.tularensis	**ABySS**	**SGA**	**SOAP2**	**SPAdes**	**SAGE**
# contigs (>= 0 bp)	133	123	289	125	148
# contigs (>= 1000 bp)	96	99	96	96	94
Total length (>= 0 bp)	1,872,907	1,782,320	1,801,735	1,802,750	1,803,179
Total length (>= 1000 bp)	1,868,305	1,773,968	1,771,846	1,794,938	1,787,585
# contigs	97	123	103	112	124
Total length	1,869,289	1,782,320	1,775,500	1,801,533	1,800,094
Largest contig	88,166	87,501	87,485	87,891	87,908
Reference length	1,892,775	1,892,775	1,892,775	1,892,775	1,892,775
GC (%)	32	32	32	32	32
Reference GC (%)	32	32	32	32	32
N50	27,975	26,517	26,805	26,876	27,942
NG50	27,727	25,217	25,184	25,906	26,867
N75	17,769	17,022	17,591	17,396	17,825
NG75	17,633	14,541	16,053	16,022	16,986
L50	24	23	23	23	22
LG50	25	25	26	25	24
L75	45	44	44	44	42
LG75	46	50	49	48	46
# misassemblies	94	35	34	28	26
Misassembled contigs length	1,279,582	545,256	604,515	480,769	467,090
# local misassemblies	46	46	47	46	56
# unaligned contigs	0 + 1 part	1 + 16 part	2 + 11 part	11 + 18 part	1 + 10 part
Unaligned contigs length	3,324	5,351	8,762	8,540	4,215
Genome fraction (%)	95.61	93.07	92.58	93.80	93.56
Duplication ratio	1.03	1.01	1.01	1.01	1.02
# N's per 100 kbp	0.64	8.98	22.70	-	15.39
# mismatches per 100 kbp	454.98	456.06	442.25	451.31	458.65
# indels per 100 kbp	63.40	65.96	85.03	58.35	72.11
Largest alignment	87,729	87,426	87,417	87,801	87,862
NA50	25,749	25,441	26,380	26,514	26,489
NGA50	25,749	23,314	23,294	23,762	23,961
NA75	15,066	15,110	16,020	16,020	16,013
NGA75	14,491	13,117	13,117	13,638	13,377
LA50	26	24	24	24	24
LGA50	26	27	27	26	26
LA75	49	47	47	46	46
LGA75	50	53	53	51	51

L.interrogans	**ABySS**	**SGA**	**SOAP2**	**SPAdes**	**SAGE**
# contigs (>= 0 bp)	184	187	418	677	118
# contigs (>= 1000 bp)	59	109	58	132	66
Total length (>= 0 bp)	4,835,775	4,587,608	4,631,026	4,698,989	4,714,477
Total length (>= 1000 bp)	4,812,190	4,560,156	4,575,322	4,585,719	4,699,920
# contigs	74	187	100	441	98
Total length	4,820,380	4,587,608	4,589,482	4,678,590	4,711,906
Largest contig	529,258	344,705	350,259	338,035	552,263
Reference length	4,277,185	4,277,185	4,277,185	4,338,762	4,277,185
GC (%)	35	35	35	35	35
Reference GC (%)	35	35	35	35	35
N50	158,414	89,452	136,042	63,637	242,370
NG50	196,390	94,110	148,198	65,245	272,239
N75	72,498	41,947	71,931	37,812	92,803
NG75	99,581	51,998	89,369	40,458	124,755
L50	8	15	10	22	7
LG50	6	13	9	20	6
L75	19	33	21	48	15
LG75	14	28	18	41	12
# misassemblies	8	2	7	114	11
Misassembled contigs length	727,575	74,491	340,426	2,871,971	1,222,667
# local misassemblies	29	42	58	159	28
# unaligned contigs	0 + 1 part	1 + 2 part	0 + 2 part	266 + 13 part	0 + 1 part
Unaligned contigs length	525,523	349,945	350,167	434,601	347,587
Genome fraction (%)	99.49	98.77	98.75	95.15	99.67
Duplication ratio	1.03	1.02	1.02	1.04	1.04
# N's per 100 kbp	26.89	66.88	209.46	42.85	-
# mismatches per 100 kbp	8.20	5.44	3.50	506.00	11.10
# indels per 100 kbp	10.81	13.45	42.36	75.17	4.48
Largest alignment	413,583	319,895	320,270	137,901	550,746
NA50	102,320	71,936	108,806	36,540	178,041
NGA50	117,711	83,128	132,993	38,735	182,864
NA75	45,117	36,142	53,558	16,344	68,620
NGA75	58,556	40,333	64,594	22,071	87,232
LA50	12	18	12	38	8
LGA50	9	16	10	34	7
LA75	29	41	26	83	20
LGA75	21	35	22	70	16

P.gingivalis	**ABySS**	**SGA**	**SOAP2**	**SPAdes**	**SAGE**
# contigs (>= 0 bp)	262	128	481	754	146
# contigs (>= 1000 bp)	87	85	73	98	68
Total length (>= 0 bp)	2,356,985	2,231,989	2,294,166	2,385,213	2,310,779
Total length (>= 1000 bp)	2,324,038	2,212,896	2,226,886	2,230,744	2,287,209
# contigs	108	128	144	573	115
Total length	2,334,502	2,231,989	2,249,473	2,369,767	2,306,842
Largest contig	190,296	171,389	184,833	154,317	196,534
Reference length	2,343,476	2,343,476	2,343,476	2,343,476	2,343,476
GC (%)	48	48	48	49	48
Reference GC (%)	48	48	48	48	48
N50	43,828	41,333	48,950	35,693	56,862
NG50	43,828	41,078	47,996	35,693	54,125
N75	23,381	21,941	24,599	15,896	27,140
NG75	22,745	18,906	22,440	17,198	26,620
L50	15	15	13	17	11
LG50	15	16	14	17	12
L75	33	34	29	42	26
LG75	34	38	32	41	27
# misassemblies	10	5	15	8	17
Misassembled contigs length	529,971	270,834	467,351	260,197	493,006
# local misassemblies	11	17	40	10	14
# unaligned contigs	0 + 0 part	0 + 0 part	0 + 1 part	423 + 0 part	0 + 0 part
Unaligned contigs length	-	-	209	115,848	-
Genome fraction (%)	97.97	95.08	95.62	95.95	97.77
Duplication ratio	1.02	1.00	1.00	1.00	1.01
# N's per 100 kbp	26.60	73.93	129.45	0.00	-
# mismatches per 100 kbp	8.84	20.28	5.44	16.59	15.62
# indels per 100 kbp	1.96	15.53	8.39	5.74	11.13
Largest alignment	172,567	167,699	167,686	154,317	172,565
NA50	35,564	40,932	47,992	32,926	54,125
NGA50	35,564	37,013	42,835	32,926	54,125
NA75	20,005	19,697	24,281	15,691	25,804
NGA75	20,005	18,062	19,982	15,716	25,176
LA50	16	15	13	18	12
LGA50	16	17	14	18	12
LA75	37	35	30	45	28
LGA75	37	39	34	44	29

E.coli	**ABySS**	**SGA**	**SOAP2**	**SPAdes**	**SAGE**
# contigs (>= 0 bp)	486	728	164	382	167
# contigs (>= 1000 bp)	71	591	76	184	73
Total length (>= 0 bp)	4,544,118	4,424,927	4,465,263	4,474,348	4,499,362
Total length (>= 1000 bp)	4,516,394	4,358,053	4,442,600	4,448,324	4,474,493
# contigs	74	728	111	215	121
Total length	4,518,213	4,424,927	4,458,606	4,463,992	4,492,807
Largest contig	329,942	54,214	326,207	162,474	327,258
Reference length	4,639,675	4,639,675	4,639,675	4,639,675	4,639,675
GC (%)	51	51	51	51	51
Reference GC (%)	51	51	51	51	51
N50	174,909	10,942	136,627	41,512	178,305
NG50	135,238	10,207	132,589	40,993	172,607
N75	79,940	6,217	70,499	22,762	73,226
NG75	74,730	5,470	60,831	20,711	67,682
L50	10	119	12	33	10
LG50	11	130	13	35	11
L75	21	256	24	68	21
LG75	22	283	26	74	22
# misassemblies	26	4	5	22	25
Misassembled contigs length	2,130,793	95,739	626,592	683,304	1,888,722
# local misassemblies	80	1	71	17	83
# unaligned contigs	1 + 0 part	4 + 7 part	1 + 8 part	2 + 3 part	2 + 5 part
Unaligned contigs length	1,490	46,118	2,825	948	4,126
Genome fraction (%)	95.62	94.10	94.80	95.09	95.42
Duplication ratio	1.03	1.00	1.02	1.01	1.06
# N's per 100 kbp	186.56	-	343.83	12.54	107.91
# mismatches per 100 kbp	11.83	8.52	6.30	15.14	11.36
# indels per 100 kbp	24.37	0.30	23.47	2.63	36.50
Largest alignment	326,073	54,214	325,634	162,291	326,332
NA50	101,741	10,611	112,232	37,047	98,856
NGA50	101,741	10,038	98,665	36,300	96,980
NA75	60,003	6,082	57,468	21,491	57,961
NGA75	56,943	5,270	54,790	18,706	54,784
LA50	13	123	13	36	13
LGA50	13	133	14	38	14
LA75	26	262	27	74	27
LGA75	28	291	30	80	29

C.thermocellum	**ABySS**	**SGA**	**SOAP2**	**SPAdes**	**SAGE**
# contigs (>= 0 bp)	3,194	436	325	2,896	354
# contigs (>= 1000 bp)	140	279	133	146	153
Total length (>= 0 bp)	3,907,516	3,563,878	3,698,354	3,746,775	3,769,337
Total length (>= 1000 bp)	3,753,091	3,492,888	3,642,276	3,578,640	3,707,091
# contigs	165	436	212	233	261
Total length	3,771,171	3,563,878	3,683,138	3,619,141	3,756,427
Largest contig	186,654	106,016	186,621	195,919	186,487
Reference length	3,843,301	3,843,301	3,843,301	3,843,301	3,843,301
GC (%)	39	39	39	39	39
Reference GC (%)	39	39	39	39	39
N50	53,679	25,255	57,028	59,462	57,180
NG50	52,944	23,747	56,876	55,150	54,883
N75	32,517	11,502	34,420	31,293	35,083
NG75	30,925	8,717	31,308	25,255	32,273
L50	20	42	19	18	18
LG50	21	47	20	20	19
L75	44	93	40	39	40
LG75	45	114	43	45	42
# misassemblies	18	1	9	6	32
Misassembled contigs length	381,996	3,070	280,458	220,044	304,372
# local misassemblies	9	-	69	6	164
# unaligned contigs	1 + 1 part	0 + 0 part	11 + 27 part	1 + 1 part	3 + 9 part
Unaligned contigs length	1,552	-	52,966	398	6,279
Genome fraction (%)	95.78	92.63	92.81	94.08	95.85
Duplication ratio	1.03	1.00	1.02	1.01	1.04
# N's per 100 kbp	49.59	-	2,097.42	6.52	98.66
# mismatches per 100 kbp	12.58	2.95	2.67	25.00	33.01
# indels per 100 kbp	1.87	0.20	70.52	3.18	13.03
Largest alignment	186,547	106,016	186,433	195,919	186,424
NA50	53,679	25,255	55,279	56,894	57,180
NGA50	52,944	23,747	54,744	52,142	54,883
NA75	30,925	11,502	31,308	31,293	32,248
NGA75	28,805	8,618	25,243	25,255	29,529
LA50	20	42	19	19	18
LGA50	21	47	20	21	19
LA75	44	93	41	40	41
LGA75	46	115	46	46	43

C.elegans	ABySS	SGA	SOAP2	SPAdes	SAGE
# contigs (>= 0 bp)	58,161	18,284	30,934	48,136	13,628
# contigs (>= 1000 bp)	10,005	8,742	6,037	8,324	6,217
Total length (>= 0 bp)	105,976,443	100,885,920	103,139,969	103,651,580	103,493,029
Total length (>= 1000 bp)	99,077,994	97,442,387	98,968,747	97,945,454	101,320,951
# contigs	17,115	18,164	9,813	15,826	9,755
Total length	102,314,962	100,861,920	100,594,200	101,072,818	102,987,712
Largest contig	230,242	240,117	386,072	183,865	383,476
Reference length	100,286,070	100,286,070	100,286,070	100,286,070	100,286,070
GC (%)	36	36	36	36	36
Reference GC (%)	35	35	35	35	35
N50	19,358	23,056	34,672	23,754	37,816
NG50	19,954	23,239	34,919	24,126	39,311
N75	8,162	9,473	16,093	10,336	16,601
NG75	8,686	9,651	16,255	10,613	18,129
L50	1,388	1,150	771	1,105	744
LG50	1,337	1,138	766	1,089	709
L75	3,415	2,865	1,827	2,703	1,774
LG75	3,235	2,820	1,813	2,647	1,657
# misassemblies	1,399	144	246	713	1,196
Misassembled contigs length	11,867,978	1,425,459	5,816,942	9,757,761	20,494,756
# local misassemblies	6,282	2,209	2,703	839	1,474
# unaligned contigs	978 + 430 part	304 + 135 part	272 + 153 part	363 + 543 part	267 + 213 part
Unaligned contigs length	4,637,025	4,615,884	4,647,857	4,698,012	4,725,891
Genome fraction (%)	95.49	95.19	95.28	95.48	96.94
Duplication ratio	1.03	1.02	1.01	1.01	1.03
# N's per 100 kbp	558.08	189.24	268.80	21.08	5.89
# mismatches per 100 kbp	24.14	6.28	10.66	59.26	26.95
# indels per 100 kbp	25.94	28.05	24.18	17.66	20.68
Largest alignment	213,835	239,959	382,096	171,314	383,476
NA50	17,721	20,311	31,886	20,245	30,897
NGA50	18,210	20,436	31,973	20,468	32,442
NA75	6,594	7,397	13,089	7,809	12,609
NGA75	7,126	7,596	13,232	8,122	14,095
LA50	1,476	1,257	835	1,287	861
LGA50	1,419	1,243	830	1,268	819
LA75	3,813	3,291	2,065	3,262	2,144
LGA75	3,591	3,233	2,047	3,187	1,992
